# On Heat Transfer Performance of Cooling Systems Using Nanofluid for Electric Motor Applications

**DOI:** 10.3390/e22010099

**Published:** 2020-01-14

**Authors:** Ali Deriszadeh, Filippo de Monte

**Affiliations:** Department of Industrial and Information Engineering and Economics, University of L’Aquila, 67100 L’Aquila, Italy; filippo.demonte@univaq.it

**Keywords:** cooling system, nanofluid, electric motor, spiral channels

## Abstract

This paper studies the fluid flow and heat transfer characteristics of nanofluids as advance coolants for the cooling system of electric motors. Investigations are carried out using numerical analysis for a cooling system with spiral channels. To solve the governing equations, computational fluid dynamics and 3D fluid motion analysis are used. The base fluid is water with a laminar flow. The fluid Reynolds number and turn-number of spiral channels are evaluation parameters. The effect of nanoparticles volume fraction in the base fluid on the heat transfer performance of the cooling system is studied. Increasing the volume fraction of nanoparticles leads to improving the heat transfer performance of the cooling system. On the other hand, a high-volume fraction of the nanofluid increases the pressure drop of the coolant fluid and increases the required pumping power. This paper aims at finding a trade-off between effective parameters by studying both fluid flow and heat transfer characteristics of the nanofluid.

## 1. Introduction

**Figure 1:**
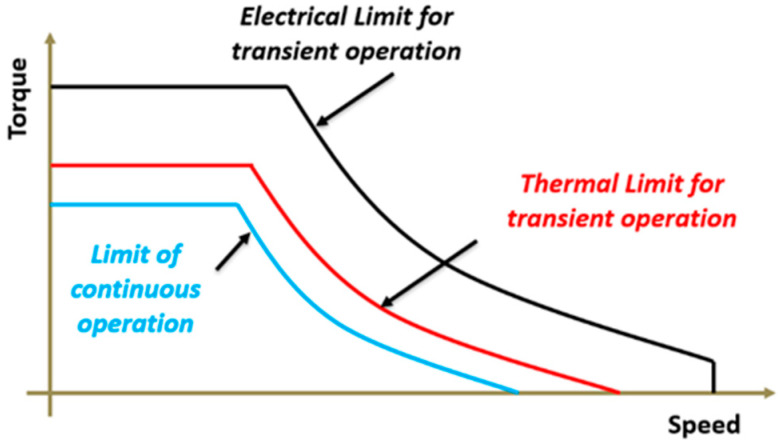
Thermal limit for transient operation of an electric motor.

As the automotive industry is on the edge of a major electrification transition, use of electric motors as the primary motive source for Electric Vehicle (EV) applications is increasingly growing [1,2]. Considering driving cycles of EVs, this new application of electric motors demands a high transition torque for accelerations [3,4]. As can be seen in Figure 1, transient operating capability of an electric motor is limited by its thermal limitations [5]. Using a high-performance cooling system, transient operating limit of an electric motor can be enhanced. Using a proper cooling system, it is possible to reduce size of electric machine for a given peak torque [6].

Considering the automotive operating environment, heat rejection capability of cooling systems of electric motors is limited by ambient temperature which is normally in the range of 50 °C to 60 °C [7]. In Hybrid Electric Vehicles (HEVs) the external ambient temperature is higher because Internal Combustion Engine (ICE) and other powertrain components such as heat exchangers release more heat to the ambient. Therefore, for vehicle application, available ambient temperature range for removing heat waste from the electric motor is narrow. This leads to need for a high-performance cooling system. Enhancing performance of a cooling system can be achieved by using advance coolant with higher heat transfer capability instead of pure water or commonly used mixture of water and ethylene glycol [8].

In general, typical cooling techniques for electric machine applications are based on air cooling systems [9,10,11], phase-change material cooling systems [12,13] and liquid cooling systems [14,15,16,17,18]. Because liquids have a higher heat transfer capacity than air of the same mass, in applications where there is a large amount of heat generated continuously, the liquid cooling systems are more suitable than air cooling systems in terms of heat transfer performance and size of the cooling system. The liquid cooling systems use a fluid (normally water or oil) as a coolant to remove the waste heat by means of convection.

As an effective way to improve heat transfer performance of the cooling system, some researches were carried out on improving heat transfer characteristics of the coolant by adding nanoparticles into the base fluid. For example, in Reference [19], for automotive radiator applications, thermohydraulic performance of a nanofluid composed of graphene and silver nanoparticles with a mixture of water and ethylene glycol as a base fluid, was studied experimentally. According to results, compare to the base fluid, the nanofluid with silver nanoparticles showed a higher increase in thermohydraulic performance coefficient than the graphene based nanofluid (average increase of up to 1.5% for graphene and 2.5% for silver). The effect of oil/SiC nanofluids on the heat transfer performance of a natural convection cooling of transformer windings was investigated numerically in Reference [20]. According to results, a significant temperature drop inside the windings of the transformer was observed (up to 10° at volume fraction of 3%). It was concluded that by increasing volume fraction of nanofluids, further improvements on heat transfer capability are observed and in some cases, the heat transfer performance is further improved by the adjusted mass flow rate distribution. The impact of nanofluids is enhanced with the rising volume fraction of nanoparticles. In this paper, Aluminum-oxide nanoparticles are selected to be added to pure water (as a base fluid). Aluminum-oxide nanoparticles are chemically stable and possess electrical insulating properties [21].

In Reference [22], using polyalphaolefin nanofluids as coolant in a laminal forced convection cooling system was studied experimentally. It was concluded that, to assess performance of nanofluids coolants, in addition to evaluate improvements in thermal conductivity, thermophysical properties of nanofluids such as viscosity must be taken into account. In other words, to make a proper evaluation of nanofluids performance as coolants, both fluid flow and heat transfer characteristics of the nanofluids must be taken into account. For this reason, in this paper, both fluid flow and heat transfer characteristics of Aluminum-oxide nanoparticles in water as a base fluid coolant are examined.

In this paper, to investigate influence of adding Aluminum-oxide nanoparticles into water on performance of the indirect cooling system is investigated by using 3D finite element method. In addition to investigating characteristics of the nanofluid, the effect of turns number of spiral pipes of the cooling jacket and the flow rate (Reynolds number) of the base coolant on heat transfer performance of the cooling system are investigated. Once the maximum heat transfer is obtained, effects of employing Aluminum-oxide nanoparticles in the base fluid on fluid flow and heat transfer performances of the nanofluid coolant are studied.

In the Section 2, boundary conditions, geometry of the electric motor and the cooling system are described. To reduce inaccuracy of the simulation study, in Section 3, pre-simulations are carried out to determine proper number of nodes in the finite element model. Governing equations are reported in Section 4. Simulation results are indicated and described in Section 5. Fluid flow and heat transfer analyses of the nanofluid are investigated in Section 6. Finally, in Section 7, conclusion of the study is presented.

## 2. Description of the Problem and Boundary Conditions

**Figure 2:**
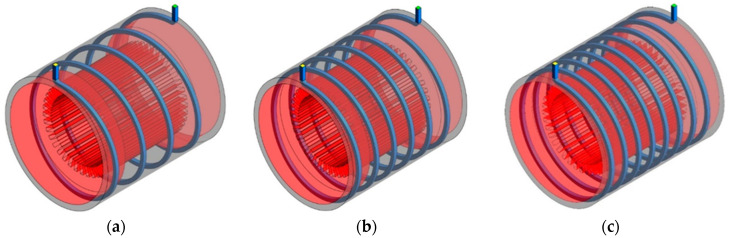
Schematic of the cooling system with (**a**) 4 turns, (**b**) 6 turns and (**c**) 8 turns.

**Figure 3:**
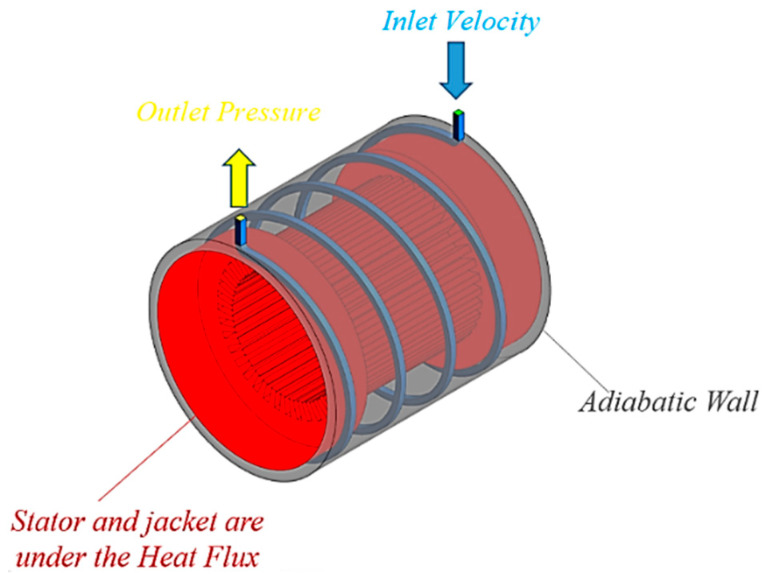
Applied boundary conditions.

Figure 2 shows schematic of the designed cooling system. The cooling system composed of a cooling jacket around the stator with spiral cooling channels. Dimensions of the electric motor and the cooling jacket are listed in Table 1. Cooling channels are made of Aluminum and material of electric motor body is steel. The wall where the channels are inside it is considered as an adiabatic wall. The coolant flows in spiral channels to remove the heat generated by the electric machine. Figure 3 shows boundary conditions. As can be seen, boundary conditions are the inlet velocity of fluid with temperature of 343 K and the outlet pressure. The generated heat flux by the 60 kw electric machine was calculated using electromagnetic analysis of the electric machine by finite element method. It was found that the stator and its cooling jacket are under heat flux of 3500 W/$m^{2}$.

The nanofluid coolant composed of Aluminum-oxide nanoparticles added to water as the base fluid. Impacts of adding three different amount of nanoparticles in the base fluid are studied to investigate the thermal performance of the cooling system according to different volume fractions.

## 3. The Problem-Solving Independency from Number of Nodes

**Figure 4:**
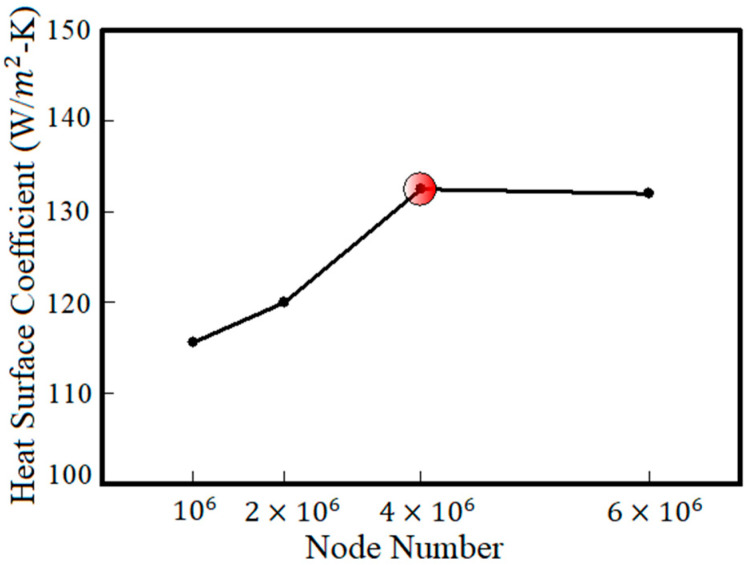
Calculated heat transfer coefficient versus number of nodes.

The accuracy of analyses can be affected by number of meshes in the problem domain. To investigate the effect of number of mesh nodes on the accuracy of obtained numerical results, different simulations were performed and average heat transfer coefficient at cooling channels wall was calculated for each simulation. Figure 4 shows the heat transfer coefficient versus number of nodes. As can be seen, as the number of nodes exceeds 6 × $10^{4}$, the heat transfer coefficient keeps not changing. Therefore, using 6 × $10^{4}$ nodes, the heat transfer coefficient can be calculated independently of number of nodes.

## 4. The Governing Equations for Heat Transfer, Calculating the Properties of Nanoparticles and Calculated Parameters

Governing equations in the Cartesian coordinates and in steady state are as follows [23].

### 4.1. Conjugation Equation

(1) $$\frac{\partial \left(u\right)}{\partial x}+\frac{\partial \left(v\right)}{\partial y}+\frac{\partial \left(w\right)}{\partial z}=0.$$

### 4.2. Momentum Equation

The Momentum equation in x-axis direction

(2) $$u\frac{\partial \left(u\right)}{\partial x}+v\frac{\partial \left(u\right)}{\partial y}+w\frac{\partial \left(u\right)}{\partial z}=-\frac{1}{\rho_{nf}}\frac{\partial P}{\partial x}+\nu_{nf}\left(\frac{\partial }{\partial x}\left(\frac{\partial u}{\partial x}\right)+\frac{\partial }{\partial y}\left(\frac{\partial u}{\partial y}\right)+\frac{\partial }{\partial z}\left(\frac{\partial u}{\partial z}\right)\right).$$

The Momentum equation in y-axis direction

(3) $$u\frac{\partial \left(v\right)}{\partial x}+v\frac{\partial \left(v\right)}{\partial y}+w\frac{\partial \left(v\right)}{\partial z}=-\frac{1}{\rho_{nf}}\frac{\partial P}{\partial x}+\nu_{nf}\left(\frac{\partial }{\partial x}\left(\frac{\partial v}{\partial x}\right)+\frac{\partial }{\partial y}\left(\frac{\partial v}{\partial y}\right)+\frac{\partial }{\partial z}\left(\frac{\partial v}{\partial z}\right)\right).$$

The Momentum equation in z-axis direction

(4) $$u\frac{\partial \left(w\right)}{\partial x}+v\frac{\partial \left(w\right)}{\partial y}+w\frac{\partial \left(w\right)}{\partial z}=-\frac{1}{\rho_{nf}}\frac{\partial P}{\partial x}+\nu_{nf}\left(\frac{\partial }{\partial x}\left(\frac{\partial w}{\partial x}\right)+\frac{\partial }{\partial y}\left(\frac{\partial w}{\partial y}\right)+\frac{\partial }{\partial z}\left(\frac{\partial w}{\partial z}\right)\right).$$

### 4.3. Energy Equation

(5) $$u\frac{\partial \left(T\right)}{\partial x}+v\frac{\partial \left(T\right)}{\partial y}+w\frac{\partial \left(T\right)}{\partial z}=\alpha_{nf}\left(\frac{\partial }{\partial x}\left(\frac{\partial T}{\partial x}\right)+\frac{\partial }{\partial y}\left(\frac{\partial T}{\partial y}\right)+\frac{\partial }{\partial z}\left(\frac{\partial T}{\partial z}\right)\right).$$

where, *u*, *v* and *w* are fluid velocities along x-, y- and z-axis, respectively. $\rho_{nf}$ is density of the nanofluid. $\alpha_{nf}$ is thermal diffusivity of the nanofluid. *T* and *P* are temperature and fluid pressure, respectively.

The fluid flow behavior and heat transfer performance of the nanofluid at different volume fractions are studied by using validated empirical relationships for thermophysical parameters such as density, specific heat capacity, thermal conductivity coefficient and the nanofluid viscosity. The used empirical equations are as follows.

(6) $$\rho_{nf}=\left(1-\varphi \right)\rho_{f}+\varphi \rho_{p}.$$

(7) $${\left(\rho C_{p}\right)}_{nf}=\left(1-\varphi \right){\left(\rho C_{p}\right)}_{f}+\varphi {\left(\rho C_{p}\right)}_{p}.$$

(8) $$\frac{k_{eff}}{k_{f}}=\frac{k_{p}+2k_{f}+2\varphi \left(k_{p}-k_{f}\right)}{k_{p}+2k_{f}-\varphi \left(k_{p}-k_{f}\right)}.$$

(9) $$\mu_{nf}=\mu_{f}\left(123\varphi^{2}+7.3\varphi +1\right).$$

where *φ*, $\rho_{f}$ and $\rho_{p}$ are volume fraction of the nanofluid, density of fluid and density of nanoparticles, respectively. *C_p_* is specific heat capacity and *K* is thermal conductivity. *K_eff_* is effective thermal conductivity. $\mu_{nf}\;$ and $\mu_{f}$ are coefficient of dynamic viscosity of nanofluid and fluid, respectively.

In calculation of the Reynolds number, the channel inlet diameter and nanofluid properties are used as follows.

(10) $$Re=\frac{\rho_{nf}u_{in}D}{\mu_{nf}}$$

In (10), *u_in_* is inlet velocity and *D* is hydraulic diameter. Equation (11) expresses the displacement heat transfer coefficient of the fluid.

(11) $$h=\frac{q_{w}}{\left(T_{w}-T_{b}\right)}$$

where *h* is heat transfer coefficient. *T_w_* and *T_b_* are wall and bulk temperatures, respectively. *q*_w_ is wall heat flux.

The following equation is used to calculate the Nusselt number.

(12) $$Nu=\frac{h\cdot D}{k_{nf}}$$

The overall thermal and fluid performance of the cooling system is evaluated using an evaluation parameter (PEC) which is defined as follows.

(13) $$PEC=\frac{\left(\frac{Nu_{ave}}{Nu_{ave,s}}\right)}{{\left(\frac{f}{f_{s}}\right)}^{\left(1/3\right)}}$$

where, *N_uave,s_* and *f_s_* are base parameters defined for the base fluid (pure water). Actually, PEC shows the ratio of the increase in heat transfer using the nanofluid coolant to the increase in the pressure drop caused by it in comparison with heat transfer and pressure drop of the base fluid.

According to empirically validated relationships, thermophysical properties of solid Aluminum-oxide nanoparticles [24] and Aluminum-oxide-Water nanofluid such as density, thermal capacity, thermal conductivity coefficient and dynamic viscosity for different volume fractions of the solid nanoparticles are listed in Table 2.

## 5. Results

**Figure 5:**
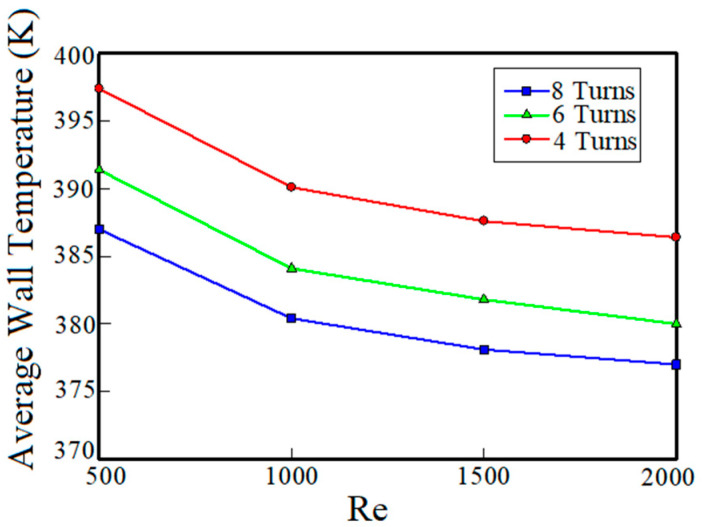
Average temperature of the motor versus Reynolds number for cooling systems using pure water coolant with different turns numbers.

The average temperature of the electric motor for different turn numbers versus the Reynolds number is shown in Figure 5 (the coolant is pure water). Results in the Figure 5 show the effect of turns number of cooling channels on heat transfer performance of the cooling system. According to Figure 5, under a constant heat flux, for all the turn numbers, as the Reynolds number increases, the average temperature of the channels’ wall decreases. For example, for the cooling system with 8 turns number and at Reynolds number of 500, the average temperature of the channels’ wall is 387 K and at Reynolds number of 2000, the average temperature is 377 K. This shows that with four times increase of the flow rate of the fluid passing through the 8 turns spiral channels, the average temperature has decreased by 10 K. This increase of the heat transfer is because of the increased heat absorption capacity due to the increase of the flow rate of the coolant fluid which leads to enhancing thermal flux absorption by the fluid and consequently the fluid temperature decreases.

In addition, from Figure 5 it can be seen that under a constant Reynolds number by increasing the turns number of cooling channels, capacity of the heat transfer from the electric motor to the coolant fluid increases. This is because of the increase in the cross-sectional area between the fluid and cooling channels. Therefore, the average wall temperature decreases. For example, for the fluid with Reynolds number of 2000, the average wall temperature of channels with 8, 6 and 4 turns numbers is 377 K, 380 K and 386 K, respectively.

**Figure 6:**
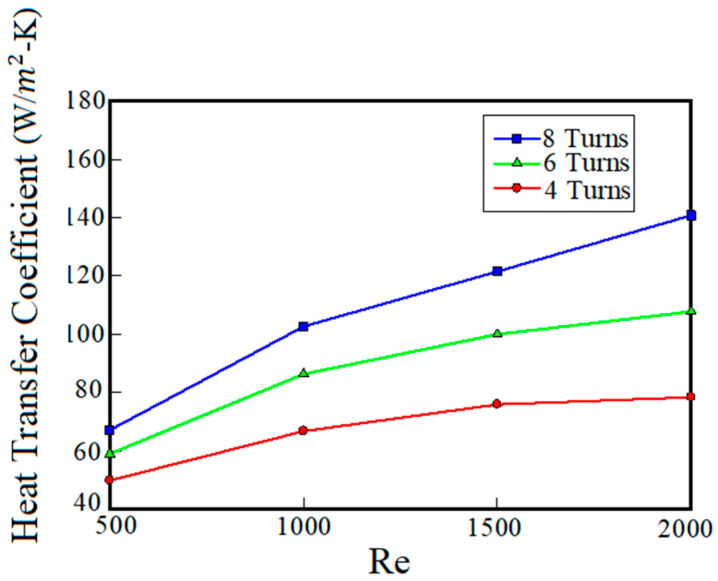
Effect of Reynolds number and turns number on heat transfer performance of the cooling system using pure water as a coolant.

Figure 6 shows the heat transfer coefficient versus Reynolds number for different turns numbers (the coolant is pure water). As can be seen in Figure 6, with the increase of the Reynolds number, because fluid flow and heat absorption capacity of channels’ walls increases, heat transfer coefficient increases. It can be seen that at 8 turns, the increase of the Reynolds number from 500 to 2000, the wall temperature decreases by 10 K. Moreover, by increasing the Reynolds number from 500 to 2000, the heat transfer coefficient increases from 67 to 140 W/m^2^ k.

According to variation of the heat transfer under different Reynolds numbers and turn numbers, it can be concluded that among the examined conditions, the best cooling performance (minimum average wall temperature) is achieved by the maximum Reynolds number and the maximum turns number. As mentioned before, in addition to thermal performance of the cooling system, it is necessary to evaluate fluid flow properties of the coolant fluid.

**Figure 7:**
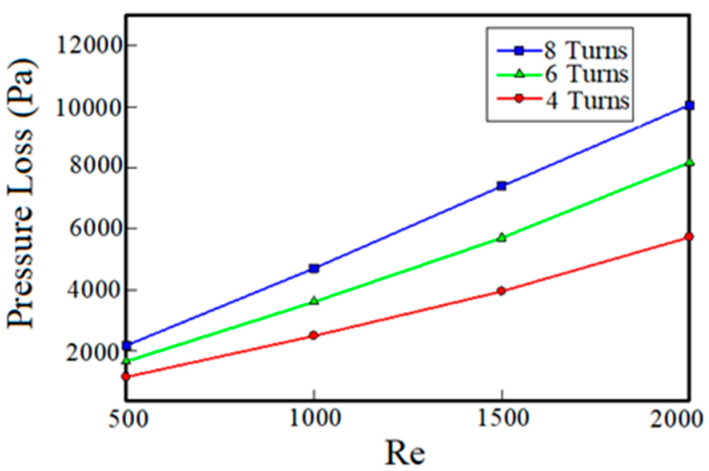
Pressure drop versus the Reynolds number for cooling systems using pure water coolant with three different turn numbers.

Figure 7 shows the pressure drop of the fluid (pure water) versus Reynolds number in different turns number of channels. As can be seen, at a given Reynolds number, the pressure drops increases with increasing turn numbers of channels. This increase of the pressure drop is because of changes in the flow path of the fluid due to the increase of turns number, the velocity gradient and the change in the arrangement of the boundary layer. By increasing the Reynolds number at constant turns number, as expected, the pressure drop has also been increased. The increase of pressure drops by increasing the Reynolds number leads to more power needed for pumping the inlet fluid flow.

## 6. Fluid Flow and Heat Transfer Analyses of The Aluminum-Oxide-Water Nanofluid

**Figure 8:**
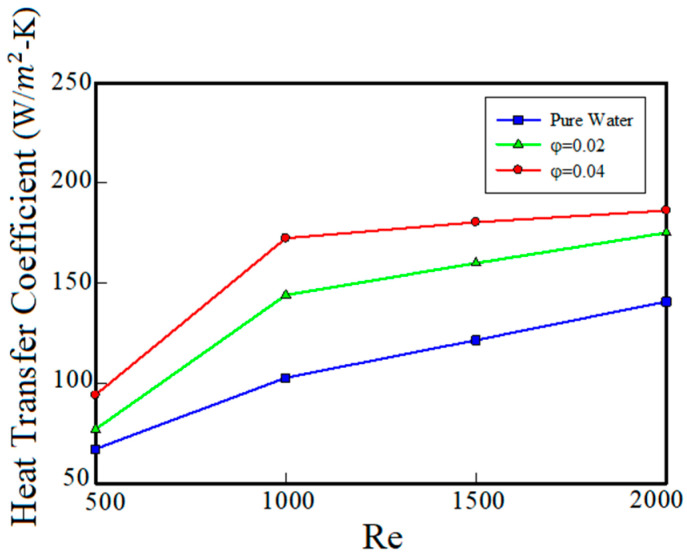
The effect of nanoparticle volume fraction on the heat transfer.

According to the results obtained for the base fluid (water), it can be concluded that highest thermal performance and the lowest operating temperature are obtained in the condition that turns number of channels is 8 and the Reynolds number is 2000. In order to increase the thermal performance of the cooling system, using a cooling jacket with 8 turns, Aluminum-Oxide nanoparticles with volume fractions of 2% and 4% are added into the base fluid. Then, fluid flow and heat transfer performances of the nanofluid are investigated. In the simulations, the nanofluid is considered as a homogeneous and single-phase fluid. Figure 8 shows the effect of nanoparticles’ volume fraction on heat transfer capability of the cooling system versus Reynolds number for the base fluid and the nanofluid with volume fractions of 2% and 4%. As can be seen, with increasing the volume fraction of nanoparticles in the base fluid, the heat transfer coefficient has increased.

According to the results, at Reynolds number of 500, by adding nanoparticles with volume fractions of 2% and 4%, the heat transfer coefficient increases by 13% and 40%, respectively. From Figure 8, it can be seen that at Reynolds number of 2000, adding nanoparticles to the base fluid with 2% and 4% volume fractions increase the heat transfer by 25% and 32%, respectively. Also, as can be seen in Figure 8, as the Reynolds number increases, the cooling capacity increases. As the Reynolds number increases, the fluid flow rate also increases. Increasing the fluid flow rate causes heat dissipation capacity to increase. Moreover, by increasing the Reynolds number, the effect of the volume fraction increasing on the heat transfer performance decreases. This means that the increased cooling capacity due to the increase of the flow rate prevails over the increased cooling capacity due to the addition of nanoparticles. For this reason, when the Reynolds number is increased, the adding of nanoparticles show less effect. But at lower fluid rates, the effect of increasing the heat transfer coefficient due to the addition of nanoparticles to the base fluid is more evident.

**Figure 9:**
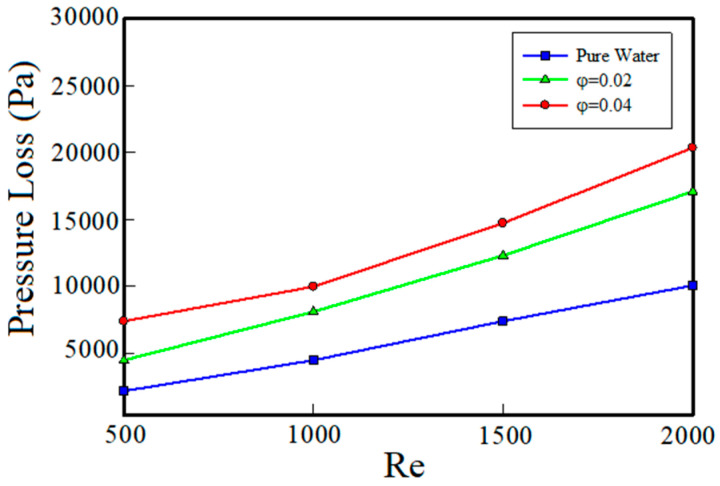
The effect of nanoparticles volume fraction on the pressure drop.

Improvement of heat transfer due to the addition of nanoparticles is because of increase in the thermal conductivity of the base fluid, which improves the heat penetration and heat transfer capability of the fluid. This improvement in heat transfer from the viewpoint of pure heat transfer is considered desirable but, along with this advantage, because of the fact that the adding nanoparticles increases the density and viscosity of the base fluid and leads to an increase in the pressure drop, it is undesirable. This explains the need to examine the effect of nanoparticles on the pressure drop. Figure 9 shows the pressure drop versus Reynolds number for three different volume fractions. As can be seen, at the same Reynolds numbers, with increasing volume fraction of nanoparticles, the pressure drop has also increased. This is because by increasing the volume fraction of nanoparticles, the viscosity of the nanofluid increases. Therefore, the cohesion force between particles of the nanofluid increases leading to increase of pressure drops. As Reynolds number increases, the effect of adding nanoparticles to the base fluid on pressure drop becomes more significant.

**Figure 10:**
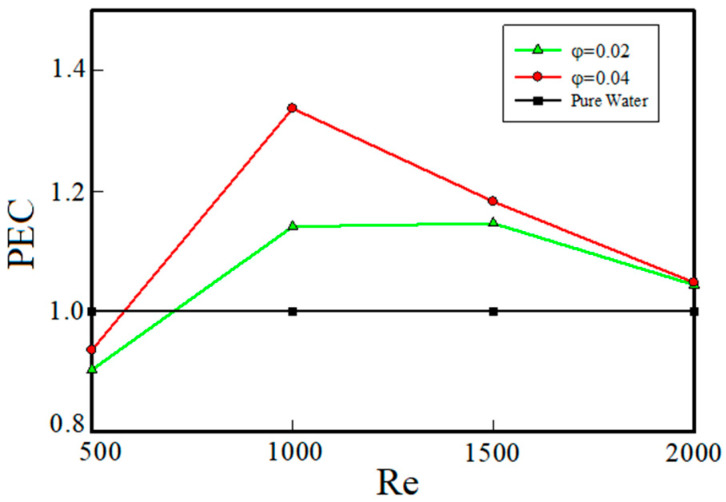
Performance coefficient versus Reynolds number.

In Figure 8 and Figure 9, the thermal and fluid flow behavior of the nanofluid were studied separately. But, for the sake of investigating the cost-effectiveness and justifying use of nanofluids as coolants from the engineering point of view, the performance of the nanofluid should be evaluated based on both fluid flow and thermal characteristics. For this purpose, the thermal-fluid flow evaluation coefficient (PEC) versus Reynolds number is shown in Figure 10 for different volume fractions. This coefficient, as defined earlier, is a measure of the rate of increase in heat transfer to the increase in the pressure drop due to the addition of nanoparticles in the base fluid. Whatever the value of this factor is greater than 1, it means that the cooling system is more cost-effective. As can be seen in Figure 10, at the minimum Reynolds number (Re = 500), the coefficient of performance is less than 1. This means that the pressure drop is dominant over the increase in heat transfer, which is not acceptable from the engineering and economic point of views. Also, according to Figure 10, with the increase of Reynolds number, the coefficient of performance has been more than 1, which indicates that the use of nanoparticles in the studied system, at Reynolds numbers of 1000 and 1500, is of desirable function and at Reynolds number of 2000, the performance coefficient of the nanofluid with volume fractions of 2% and 4% is approximately the same. Therefore, at Reynolds number of 2000, it is beneficial to use a nanosilver with 2% volume fraction because volume fraction of 4% leads to a much greater pressure drops. When Reynolds number is 1000, PEC has the highest value. This is because in this Reynolds number, the increase in heat transfer coefficient overcomes the increase in pressure drop. But as the Reynolds number increases, the pressure drop increases more than the increase in heat transfer coefficient.

**Figure 11:**
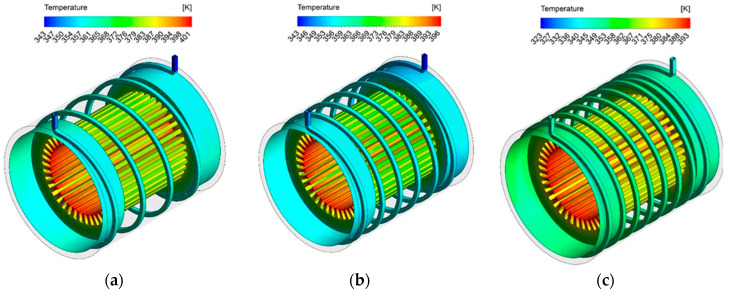
Temperature distribution contours of the cooling system using the nanofluid at Reynolds number of 2000 with (**a**) 4 turns, (**b**) 6 turns and (**c**) 8 turns.

So far, the results are presented quantitatively. In this section, in order to study the flow quality, contour of temperature distribution is presented in various conditions. These contours show the effect of the Reynolds number, the turns number of cooling channels and the volume fraction of nanoparticles on the heat transfer performance and the reduction of the electric motor operating temperature. In Figure 11, contours of temperature distribution for three different turns numbers at Reynolds number of 2000 are shown.

As can be seen in Figure 11, the temperature of the electric motor has been increased due to the production of stator thermal flux and the temperature of the coolant fluid that flows through the spiral channels, as it approaches the outlet, is brought about by temperature rise. Investigating the effect of turns numbers shows that with the increase in the turns number of cooling channels, due to the increase of the cross-section in contact with the nanofluid, the maximum operating temperature has decreased.

**Figure 12:**
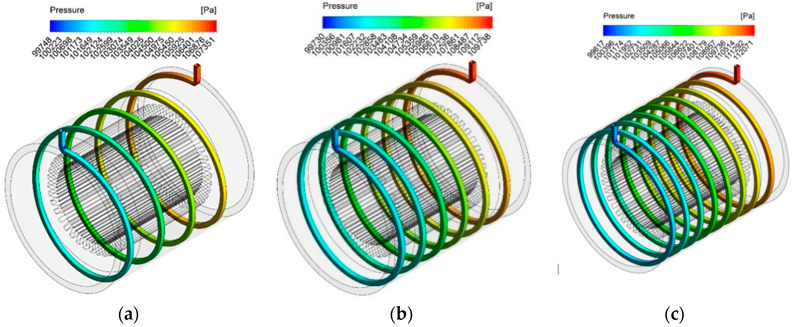
Pressure distribution contours of the cooling system using the nanofluid with different turns numbers (**a**) 4 turns, (**b**) 6 turns and (**c**) 8 turns.

Figure 12 shows the contours of the pressure distribution inside the spiral channels with different turns numbers. As can be seen, with the increase in the number of turns, the pressure drop also increases due to the velocity gradient and the change in the flow field along the spiral channels.

**Figure 13:**
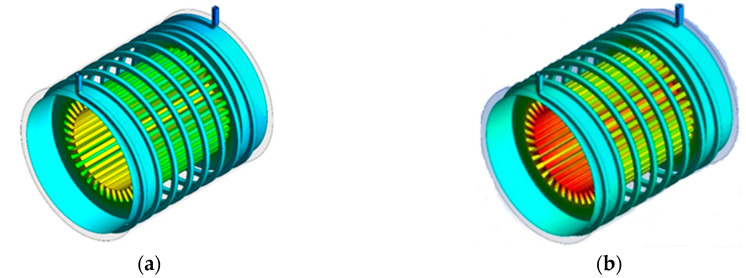
Temperature distribution contours at Reynolds number of 2000 with volume fractions of (**a**) 2% and (**b**) 4%.

In Figure 13, contours of temperature distribution are shown at the Reynolds number of 2000 for the nanofluid with volume fractions of 2% and 4%. As can be seen, by increasing the volume fraction of nanoparticles in the fluid, the heat transfer from channel’s walls to the cooling fluid increases and the motor temperature decreases. The improvement in cooling due to the use of nanoparticles in the base fluid results from the fact that the addition of nanoparticles to the base fluid increases its conduction heat transfer coefficient. This improves thermal penetration depth of the coolant fluid. Also, the presence of a nanoparticle in the base fluid leads to a reduction in specific heat capacity of the base fluid. This increases amount of the absorbed heat flux by the coolant fluid. These two factors reduce the operating temperature of the motor and improve heat transfer performance of the cooling system.

## 7. Conclusions

Using the finite element numerical method, a cooling system of an electric motor has been simulated. In order to increase the heat transfer capability of the cooling system and to reduce operating temperature of the electric motor, a nanoparticle of Aluminum-oxide has been added to the base fluid (pure water). The effect of nanoparticle volume fraction on the fluid flow and heat transfer properties of the nanofluid coolant has been investigated. Also, the effect of the flow rate of the cooling fluid and turns number of spiral channels on heat transfer capability of the cooling system have been studied. The studied results include heat transfer coefficient, pressure drop and average temperature. The numerical results show that with increasing Reynolds number, the operating temperature of the electric motor decreases and the pressure drop increases. According to the simulation results, adding nanoparticles with volume fraction of 4%, increases the heat transfer capability of the cooling system up to 40%. The performance coefficient test shows that the increase in the pressure drop due to the addition of nanoparticles in fluids with a low Reynolds number (500) is significand while increase in heat transfer capability is very limited.

Also, in Reynolds number of 2000, addition of nanoparticles with volume fraction of 2% and 4% almost lead to a same performance. Therefore, it can be concluded that by considering thermal and fluid performance of the system with Reynolds number of 2000, the use of Aluminum-oxide nanoparticle with 2% volume fraction shows better heat transfer performance and is more cost-effective. Finally, according to the results, in case of using fluid with Reynolds number of 1000 for both volumetric 2% and 4% and with channel turns number of 8, improvement in heat transfer capability is more remarkable compare to the increase of pressure drop.

## Figures and Tables

**Table 1:** Dimensions of the electric motor and the cooling jacket.

	Cooling Jacket	Electric Motor
**Length**	190 mm	210 mm
**Length**	240 mm (with coils)	240 mm
**Thickness**	____	10 mm

**Table 2:** Characteristics of Aluminum-oxide nanoparticles for different volume fractions [24].

φ (%)	ρ (J/kg K)	Cp (kg/m^3^)	k (W/m K)
φ = 0	998.2	4182	0.613
φ = 0.02	1056.6	3922.4	0.6691
φ = 0.04	1116	3693.2	0.7296
Al_2_O_3_	3970	765	40
